# Representation of less-developed countries in Pharmacology journals: an online survey of corresponding authors

**DOI:** 10.1186/1471-2288-11-60

**Published:** 2011-05-05

**Authors:** Dileep K Rohra

**Affiliations:** 1 Department of Pharmacology, Alfaisal University, Riyadh, Kingdom of Saudi Arabia

## Abstract

**Background:**

Scientists from less-developed countries (LDC) perceive that it is difficult to publish in international journals from their countries. This online survey was conducted with the primary aim of determining the opinion of corresponding authors of published papers in international Pharmacology journals regarding the difficulties in publications and their possible solutions.

**Methods:**

The titles of all Pharmacology journals were retrieved from Pubmed. 131 journals were included in study. The latest issue of all journals was reviewed thoroughly. An online survey was conducted from the corresponding authors of the published papers who belonged to LDC.

**Results:**

584 out 1919 papers (30.4%) originated from the LDC. 332 responses (response rate; 64.5%) were received from the authors. Approximately 50% the papers from LDC were published in journals with impact factor of less than 2. A weak negative correlation (r = -0.236) was observed between journal impact factor and the percentage of publications emanating from LDC. A significant majority of the corresponding authors (n = 254; 76.5%) perceived that it is difficult to publish in good quality journals from their countries. According to their opinion, biased attitude of editors and reviewers (64.8%) is the most important reason followed by the poor writing skills of the scientists from LDC (52.8%). The authors thought that well-written manuscript (76.1%), improvement in the quality of research (69.9%) and multidisciplinary research (42.9%) are important determinants that may improve the chances of publications.

**Conclusions:**

The LDC are underrepresented in publications in Pharmacology journals. The corresponding authors of the published articles think that biased attitude of the editors as well as the reviewers of international journals and the poor writing skills of scientists are the major factors underlying the non-acceptance of their results. They also think that the improvement in the writing skills and quality of research will increase the chances of acceptance of their works in international journals.

## Background

Publishing in prominent scientific journals provides better visibility and impact of research results at individual as well as institutional and national level. Scientists are under continuous pressure to publish in international journals in order to obtain rewards and promotions [1,2]. This also applies to scientists residing in less-developed (middle and low income) countries. Scientists from less-developed countries (LDC) perceive that it is difficult to get publications in reputable biomedical journals and the results of the studies in the fields of epidemiology [3], psychiatry [4], and cardiology [5] confirm the underrepresentation of LDC authors in the respective international journals. The conclusion of one survey indicates that researchers from LDC believe that the editorial bias against their works based on geographical location is a major reason for underrepresentation in publications [6].

In addition to quantity, the quality is increasingly recognized as a critical aspect while evaluating research. In the 1970s, some objective parameters were suggested as a means to evaluate quality of research [7,8]. Among these parameters, the impact factor (IF) of the journal is widely employed as a tool to judge the quality of scientific research [9,10]. The idea of IF was first coined by Garfield in 1955 [11]. It is an instrument for the assessment of quality of the journal monitored periodically by Thomson Scientific (formerly International Scientific Institute-ISI), Philadelphia [12]. Although, there are several criticisms regarding the usage of IF as a tool to measure the quality of publication [13,14], it is still a very simple, convenient and quick indicator for assessing the impact and quality of research [15]. Furthermore, this parameter is given a lot of weightage for recruitments, funding, promotions and rewards [15].

To date, no study has documented the opinion of scientists from LDC regarding the difficulties in publishing their work. Therefore, this online survey was conducted with the primary aim of determining the opinion of corresponding authors of published papers in international Pharmacology journals regarding the perceived difficulties in publishing their findings and the possible solutions. The secondary aim of the study was to determine the number of publications emanating from LDC in Pharmacology journals.

## Methods

### Identification and inclusion of journals

Pubmed was accessed in the first week of June 2010. Medline journals were searched by the broad subject term "Pharmacology", while related subject terms were not taken into account. The search generated 297 titles of Pharmacology journals which were indexed by Pubmed. After a thorough scrutiny, 131 journals were included in study (Table 1). The rest were excluded due to one of the following reasons. The journal was no longer being published with the same name as was retrieved from search; this included change of name or the merger with some other journal, or the last publication was more than a year old. Non-English language journals were also excluded from the study.

**Table 1:** Journals included in the survey.

AAPS J	Drug Discov Today	J Pharmacol Sci
AAPS PharmSciTech	Drug Metab Dispos	J Pharm Pharm Sci

Acta Pharmacologica Sinica	Drug Metab Pharmacokinet	J Physiol Pharmacol

Adv Drug Delivery Rev	Drug Metab Lett	J Vet Pharmacol Ther

Adv Pharmacol	Drug Metab Rev	Magnes Res

Aliment Pharmacol Ther	Drug News Perspect	Mar Drugs

Ann Pharmacother	Drug Test Anal	Med Res Rev

Arzneimittel-Forschung	Drugs	Methods Find Exp Clin Pharmacol

Assay Drug Dev Techn	Eur J Clin Pharmacol	Mol Diagn Ther

Auton Autacoid Pharmacol	Eur J Pharm Sci	Mol Interv

Basic Clin Pharmacol Toxicol	Eur J Pharm Biopharm	Mol Pharm

Behav Pharmacol	Eur J Pharmacol	Mol Pharmacol

Biochem Pharmacol	Eur Rev Med Pharmacol Sci	Nat Prod Commun

Biol Pharm Bull	Expert Opin Drug Deliv	Nat Rev Drug Discov

Biopharm Drug Dispos	Expert Opin Drug Saf	Naunyn-Schmiedeberg's Arch Pharmacol

Brit J Clin Pharmacol	Expert Opin Pharmacother	Neuropharmacology

Brit J Pharmacol	Expert Rev Pharmacoeconomics Out Res	Pak J Pharm Sci

Can J Physiol Pharmacol	Food Drug Law J	PDA J Pharm Sci Technol

Cardiovasc Hematol Agents Med Chem	Fund Clin Pharmacol	Pharm Dev Technol

Cell Physiol Biochem	IDrugs	Pharm Res

Cent Nerv Syst Agents Med Chem	Immunopharmacol Immunotoxicol	Pharm Stat

Chem Pharm Bull	Indian J Physiol Pharmacol	PharmacoEconomics

Chem Biol Drug Des	Inflammopharmacology	Pharmacogenet Genomics

Chem-Biol Interact	Int Immunopharmacol	Pharmacogenomics

ChemMedChem	Int J Clin Pharmacol Ther	Pharmacogenomics J

Clin Drug Invest	Int J Immunopathol Pharmacol	Pharmacol Rep

Clin Exp Pharmacol Physiol	Int J Pharm	Pharmacol Research

Clin Pharmacokinet	Invest New Drugs	Pharmacol Reviews

Clin Pharmacol Ther	J Basic Clin Physiol Pharmacol	Pharmacology

CNS Drugs	J Biopharm Stat	Pharmacol Ther

Comp Biochem Physiol C	J Cardiovasc Pharmacol	Pharmacol Biochem Behav

Curr Clin Pharmacol	J Cardiovasc Pharmacol Ther	Proc West Pharmacol Soc

Curr Comput Aided Drug Des	J Clin Pharmacol	Prog Drug Res

Curr Drug Deliv	J Control Release	Prog Med Chem

Curr Drug Discov Technol	J Drug Target	Pulm Pharmacol Ther

Curr Drug Saf	J Ethnopharmacol	Recent Pat Drug Deliv Formul

Curr Mol Pharmacol	J Nat Prod	Recent Pat Inflamm Allergy Drug Discov

Curr Opin Drug Discov Dev	J Neuroimmune Pharmacol	Regul Toxicol Pharmacol

Curr Opin Pharmacol	J Ocul Pharmacol Ther	Skin Pharmacol Physiol

Curr Pharm Biotechnol	J Pharm Biomed Anal	Toxicol Appl Pharmacol

Curr Pharm Des	J Pharm Pharmacol	Trends Pharmacol Sci

Curr Vasc Pharmacol	J Pharm Sci	Value Health

Drug Deliv	J Pharmacol Exp Ther	Vasc Pharmacol

Drug Des Dev Ther	J Pharmacol Toxicol Methods	

The titles of the journals were retrieved from Pubmed using key word "Pharmacology". After exclusion as mentioned in "Methods", all journals mentioned in the Table were analyzed in the study.

### Data collection

**Figure 1:**
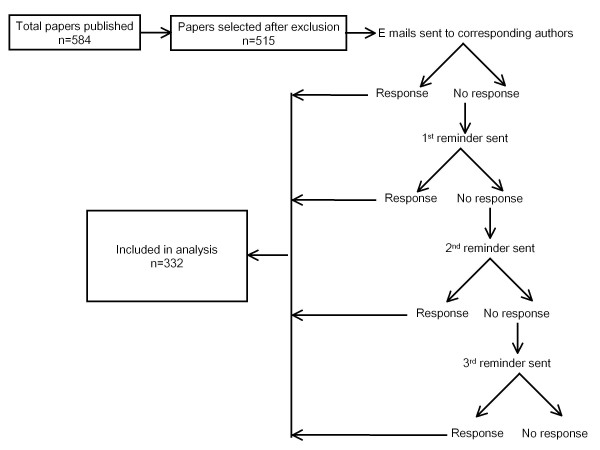
**Flow chart of the study**.

The latest issue of each of the selected 131 Pharmacology journals was reviewed thoroughly. For this, the web sites of the journals were accessed to check for the latest print issue. For online only journals, the latest issue was taken into account. The web sites of certain journals were non-functional; in those cases the abstracts of the latest issues were retrieved from Pubmed. The addresses of corresponding authors of original articles including short communications, and review papers were obtained. Editorials, commentaries and correspondences were excluded. The articles were selected in which the corresponding authors belonged to countries other than the high income OECD countries. At the time of designing the study (April 2010), 27 countries were mentioned as members of the OECD on the Organisation's web site and accordingly these countries were excluded from the study. A data base was created in Microsoft word for those articles published from non-OECD countries. The data base contained abstract with full citation data, names of the authors with affiliation and e mail addresses of corresponding authors (if available). A review of the latest issues of journals was started from the last week of June 2010 and was completed in the middle of September. A separate data base was created in Microsoft Excel in which the information about the volume and issue of the journals (which were reviewed), date of publication, journal IF for 2009, total number of articles published in that particular issue, number of papers from non-OECD countries and the country of corresponding authors were entered. All the corresponding authors whose e mail addresses were available were contacted through e mail. A structured questionnaire was sent to them that contained questions asking their opinion regarding the publications from the LDC in Pharmacology journals (Appendix I). In a few instances, there was more than one paper from the same corresponding author in one issue; in that case separate e mails were sent to them for each paper. In some papers, there were more than one corresponding authors; in that case, all corresponding authors were contacted. The first response for that particular publication was entered for analysis. For non-responders, the reminders (up to three) were sent after two weeks following the preceding e mail. Figure 1 depicts the flow of study.

### Statistical analysis

All the filled questionnaires from the corresponding authors were edited, coded and the responses were entered. As per the objectives of the study, the data presented are mainly descriptive. Pearson Correlation Coefficient was used to observe the direction and strength of relationship between the journal IF and percentage of publications emanating from the LDC. A scatter plot was also made to demonstrate this relationship.

## Results

As mentioned in the Methods, after exclusion, 131 Pharmacology journals were reviewed (Table 1). The total number of papers in all journals was 1919. Out of these, 584 (30.4%) originated from the non-OECD (hereafter referred as less-developed) countries. E mail addresses of 69 corresponding authors could not be retrieved. Corresponding authors of the remaining 515 papers were sent an e mail with an attached questionnaire. E mail could not be delivered to nine authors and five authors declined to participate. At the end of the study, 332 responses (response rate; 64.5%) were received (Figure 1).

The characteristics of the papers published in Pharmacology journals and those of the authors are presented in Table 2. Approximately half of the papers from LDC were published in journals with an IF of less than 2. Only 7.0% of all the papers were published in journals with an IF of more than 4. As mentioned by the corresponding authors, 47.9% of those publications had been rejected previously by other journals. The average number of times, the currently published papers were earlier rejected was 1.57 (range; 0-4). Nearly 8% of the publications contained at least one coauthor from countries belonging to OECD. An overwhelming majority of the corresponding authors were faculty (86.2%) followed by students (6.6%).

**Table 2:** Characteristics of papers and their corresponding authors.

Characteristics	n (%) N = 584*	Characteristics	n (%) N = 332*
Journal Impact Factor		Paper rejected from other journal(s)	159 (47.9)
<1.00	165 (28.2)		
1.00-<2.00	118 (20.2)		
2.00-<3.00	186 (31.9)		
3-<4	73 (12.5)		
≥4	42 (7.2)		
Number of authors		Corresponding authors	
1	12 (2.1)		
2-4	242 (41.4)	Faculty	286 (86.2)
5-7	226 (38.7)	Students	22 (6.6)
8-10	81 (13.9)	Industry	14 (4.2)
>10	23 (3.9)	person	10 (3.0)
		Others	
Co-author(s) from developed country	46 (7.9)		

*The number of responses varies because the source of data as mentioned above in the Table caption is different.

The table shows the characteristics of the publications and the corresponding authors. The data on the number of authors and the presence of coauthor from developed countries was obtained from the papers themselves. The data on previous rejection(s), and position of corresponding authors were obtained from the authors themselves via e mail.

**Figure 2:**
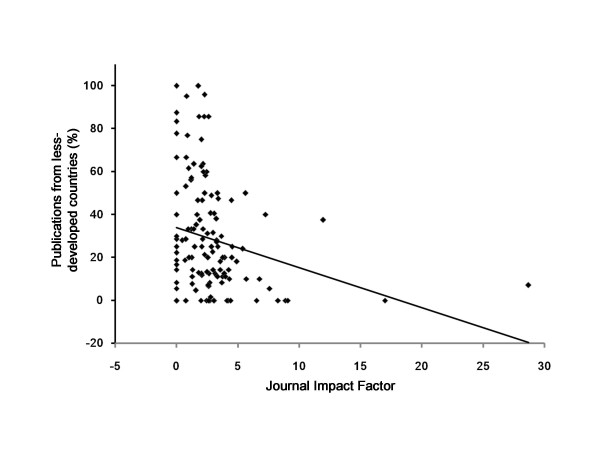
**Correlation between the journal impact factor and the publication rate**. The scatter plot shows a weak negative correlation between the impact factor of the journal and publications (in percentage) from the less-developed countries. The trendline shows that as the impact factor of the journal increases the publication rate from the less-developed countries decreases.

The journal IF for year 2009 was noted. Figure 2 illustrates the correlation between the journal IF and the publication rate from the LDC. A weak negative correlation (r = -0.236) was observed between the journal IF and the percentage of publications emanating from LDC. This indicates that as the IF of the journal increases the percentage of publications from LDC decreases.

Table 3 shows the country affiliation of corresponding authors. Forty-three countries were represented in the papers published in Pharmacology journals. China had the highest number of publications (28.6%) followed by India (19.7%) and Brazil (6.3%).

**Table 3:** Major countries contributing to publications from developing countries

Country	Number of publications (% of total)
China	167 (28.6)

India	115 (19.7)

Brazil	37 (6.3)

Korea	36 (6.2)

Mexico	35 (6.0)

Poland	30 (5.1)

Iran	20 (3.4)

Turkey	15 (2.6)

Argentina	14 (2.4)

Others (34 countries)	115 (19.7)

**Total**	**584 (100.00)**

A significant majority of the corresponding authors (n = 254; 76.5%) perceived that it is difficult to publish in good quality journals from their countries. These authors who perceived difficulties in publication from their countries were further asked to identify the reasons underlying the perceived difficulties in publication. 250 authors responded to this question and according to their opinion, biased attitude of editors and reviewers (64.8%) is the most important reason, followed by poor writing skills of the scientists (52.8%) from LDC (Table 4).

**Table 4:** Reasons underlying difficulties in publications in the opinion of corresponding authors.

Reason	n (% of respondents) N = 250
Biased attitude of reviewers and editors against authors from developing countries	162 (64.8)

Poor writing skills	132 (52.8)

Reviewers and Editors do not trust data generated in developing countries	100 (40.0)

Quality of research is not good in developing countries	78 (31.2)

Lack of funding and other resources	74 (29.6)

Unimpressive publication record of the authors	42 (16.8)

Lack of generalisibility of results	18 (7.2)

Total responses*	**606**

*The number of responses (606) exceeds the number of respondents (250) because the majority of respondents selected more than one reasons.

The corresponding authors who thought that publishing from their countries is difficult were asked to identify the reasons underlying the difficulties in publishing.

All corresponding authors were also asked to identify the factors which might enhance the chances of acceptance of papers submitted by scientists from LDC. As shown in Table 5, according to their opinion, well-written manuscript (76.1%), improvement in the quality of research (69.9%) and multidisciplinary research (42.9%) are important determinants that may improve the chances for publications.

**Table 5:** Attributes that increase the chances of acceptance for publication in good quality journals.

Attribute	n (% of respondents) N = 326
Well-written manuscript	248 (76.1)

Improvement in the quality of research	228 (69.9)

Multidisciplinary research	140 (42.9)

Having collaborator/coauthor from developed country	120 (36.8)

Sound publication record	86 (26.4)

Total responses*	**822**

*The number of responses (822) exceeds the number of respondents (326) because majority of respondents selected more than one attribute.

All corresponding authors from the less-developed countries were asked to identify the measures that can increases the chances of acceptance of their manuscript.

## Discussion

This study has documented for the first time the representation of papers from LDC in Pharmacology journals and the opinion of the corresponding authors from these countries regarding the difficulties in publishing their studies.

The classification of the countries into developed and less-developed was based upon the membership of OECD. Those with OECD membership were labeled as developed and others as less-developed. The rationale for following this classification is that as OECD membership is restricted to high income countries as defined by the World Bank [16] which have an established democratic government set up [17]. However, it should be noted that at the time of the study design, the membership of OECD was only 27, which has expanded up to 34 countries at the time of writing the manuscript. The data obtained from the authors of 7 new OECD members is included with other LDC.

According to US Census Bureau's International Data Base, OECD countries comprise only 13% of the world population in the year 2010 [18]. However, this part of the world contributed more than two thirds of the total number of publications in Pharmacology journals compared to around 30% by the LDC. There were four journals; one each from China, India, Pakistan and Poland which published articles mainly from their own countries. This somewhat inflated the representation of publications from LDC. If we exclude these four journals from the analysis, the actual representation in international journals drops to 26.7%. Previously, publication rates of 4.8 [19] and 6.0% [4] in psychiatry, 5.0% in tropical medicine [20], 6.5% in internal medicine [21], 5.5% in ophthalmology [22], 0.3% in anesthesiology [23] and very few in surgery [24] journals from LDC have been reported. These figures are grossly different from those presented in this study. However, there is a basic difference in the methodology of above studies and the current one. Previous reports have analysed only those papers that were published in leading journals with high IF in their respective fields. However, our study has analysed all Medline-indexed Pharmacology journals. Since we found that the publication rate from LDC is weakly but negatively correlated with the IF of the journal, the top ten highest ranking journals were analysed, separately. As expected, the publication rate from LDC markedly dropped to 10.2% in those journals, which approaches to already published reports. This finding is consistent with the response of corresponding authors; an overwhelming majority of whom believes that it is difficult to publish in high quality international journals.

In the face of the population size of the LDC, there is a gross underrepresentation of publications emerging from these countries. In order to understand the reasons underlying this underrepresentation, the corresponding authors were asked to identify them. Broadly, the reasons identified by them can be grouped into two categories. First, the reasons that are associated with the review process of the journals such as biased attitude and lack of trust among reviewers and editors on the data generated in their countries. In the second category, the reasons are associated with deficiencies prevailing within the scientists from LDC such as poor writing skills and poor quality of research. The former reasons were identified as the major hurdles underlying the non-acceptance of their publications by an overwhelming majority of authors. Indeed, there are some reports that have suggested that the data generated in low income nations is undervalued [25]. Moreover, a statistically significant difference between the high and low income countries in terms of rejection rate of submitted manuscripts has been shown [26].

As far as the improvement in the quality of research in LDC is concerned, this is mainly linked to the political system of the country. It is known that research is the last priority in the majority of LDC with science being considered by governments as a luxury that can be afforded only by rich countries [27]. Thus poor funding, lack of incentives to scientists and resources will eventually lead to poor research facilities, limited technical support and inadequate training. Obviously, with all these barriers, the scientific activities and research will be hampered. It is conceivable that under these less-encouraging circumstances, both quantity and quality of research would also be affected. Indeed, it has been shown that the number of submissions from LDC was far fewer (5.2% of the total submissions) compared to high income countries in psychiatry journals [19]. As far as the other reasons identified by the authors are concerned, if these are valid, they can easily be resolved by the scientific community and the international journals. For example, one inherent reason is the poor writing skills of researchers from LDC because English is not the first language for the majority of these nations. Influential international journals with adequate human resources can come forward and offer a free service for checking and editing the manuscripts from non-English speaking scientists once they are accepted for publication. As a matter of policy, rejection of papers based on the inadequate usage of language should not be acceptable. International journals can initiate another measure to bridge this gap between the developed countries and LDC by reserving some pages for papers from LDC. This reserved section should publish the best research emanating from the LDC only. This would have a positive impact on the enthusiasm of the scientists. One case can be cited to reinforce this suggestion. The Journal of Pakistan Medical Association started a reserved section for students in 2004. This fostered an interest towards research among undergraduate medical students and student research groups at regional and national levels were made. Publications of their results as lead authors motivated them to involve themselves in larger projects. Recently, the undergraduate medical students have launched their own journal. Furthermore, notable scientists from the LDC should be invited to serve on the editorial staff of the international journals since presently, the majority of editorial board members of international medical journals are residents of highly developed nations [20,28]. This step may help eliminate the feeling among authors from LDC that their findings are not given importance.

One limitation of the study is that the data was generated from papers published in only one issue of all Pharmacology journals. It is likely that these data may not be representative of the overall situation. However, the inclusion of all the journals in the current study has yielded a sample size which may be considered as sufficiently representative. Second limitation of the current study is that division of countries is based on the membership of OECD. This has put some high income countries like oil-rich Gulf States into LDC. However, this has been done purposefully because the academic history of these countries is rather brief. The third limitation is that Pharmacology related subject terms like antibacterial drugs, toxicology etc were not taken into account resulting in many journals that were not included.

## Conclusions

The LDC are underrepresented in publications in Pharmacology journals. The corresponding authors of the published articles perceive that biased attitude of the editors as well as the reviewers of international journals and the poor writing skills of scientists are the major factors underlying the non-acceptance of their scientific work. They also think that the improvement in the writing skills and quality of research will increase the chances of acceptance of their works in international journals.

## Competing interests

The author declares that they have no competing interests.

## Appendix I

Q1. You are a

□ Faculty

□ Industry person

□ Trainee

□ Support staff

□ Student

□ Any other (please specify)

Q2. Did you submit the present paper in some other journal(s) where it was rejected?

□ Yes

□ No

Q3. How many journals?

□ 1

□ 2

□ 3

□ 4

□ More than 4

Q4. How long did it take from drafting of manuscript to final acceptance (including all the previous rejections if any)? (months)

Q5. Do you think that it is difficult to publish papers from your country in journals of high impact factors?

□ Yes

□ No

Q6. If your answer to question 5 is yes than what do you think might be the reason(s) (you can choose more than one option)?

□ Quality of research is not good

□ Biased attitude of editors and reviewers towards researchers from developing countries

□ Writing skills of scientists from developing countries are not good

□ Editors & reviewers do not trust data from developing countries

□ Lack of generalisibility of results from developing countries

□ Unimpressive publication record of the authors

□ Lack of funding to cover publication costs in some journals

□ Any other (please specify)

Q7. What in your opinion will increase the chances of acceptance in journals of high impact factors (you can choose more than one option)?

□ Improvement in the quality of research

□ Well written manuscript

□ Multidisciplinary research

□ Having co-author from developed country

□ Sound publication record of first or corresponding author

□ Any other (please specify)

## Pre-publication history

The pre-publication history for this paper can be accessed here:

http://www.biomedcentral.com/1471-2288/11/60/prepub
